# Chemical Composition, Nutritional Value, and Safety of Cooked Female *Chaceon* *Maritae* from Namibe (Angola)

**DOI:** 10.3390/foods8070227

**Published:** 2019-06-26

**Authors:** Celso Manuel Cristovão Mandume, Narcisa M. Bandarra, Joana Raimundo, Helena Maria Lourenço, Susana Gonçalves, Marta Ventura, Inês Delgado, Andreia Rego, Carla Motta, Isabel Castanheira, Maria Leonor Nunes, Maria Paula Duarte

**Affiliations:** 1 MEtRICs/DCTB, Faculdade de Ciências e Tecnologia, Universidade NOVA de Lisboa, 2829-516 Caparica, Portugal; 2 IPMA—Instituto Português do Mar e da Atmosfera, R. Alfredo Magalhães Ramalho 6, 1495-165 Algés, Portugal; 3 CIIMAR, Universidade do Porto, Terminal de Cruzeiros do Porto de Leixões, Avenida General Norton de Matos, S/N 4450-208 Matosinhos, Portugal; 4 INSA-Instituto Nacional de Saúde Doutor Ricardo Jorge, Av. Padre Cruz, 1600-407 Lisboa, Portugal

**Keywords:** *Chaceon maritae*, muscle, hepatopancreas, ovaries, fatty acids, amino acids, mineral composition, toxic elements, nutritional quality, food analysis

## Abstract

Despite being highly appreciated and consumed, the nutritional value of *Chaceon maritae* from Namibe (Angola) had never been studied. In the present work, edible tissues (muscle, ovaries, and hepatopancreas) of boiled female *C. maritae* caught off Namibe coast in two distinct seasons were analyzed in terms of proximate chemical composition (fat, ash, protein, and moisture), fatty acid and amino acid profiles, cholesterol, essential minerals (macro and trace) and toxic elements. Results showed that, in both seasons, *C. maritae* muscle was a valuable source of protein, essential amino acids, polyunsaturated fatty acids, and essential elements, especially zinc, selenium, iodine, and copper. Ovaries and hepatopancreas are also good sources of protein, but were richer in fat, particularly when caught in October. Ovarian fat is rich in polyunsaturated fatty acids and that of hepatopancreas has higher values of monounsaturated and saturated fatty acids. Hepatopancreas and ovaries are also good sources of copper and, especially ovaries, of zinc. Moreover, in both seasons, all the edible tissues of *C. maritae* analyzed presented very low contents of heavy metals (mercury, cadmium, lead, and arsenic).

## 1. Introduction

The deep-sea red crab *Chaceon maritae*, originally described as *Geryon maritae* [1], belongs to Geryonidae family, which comprise several species distributed worldwide, except for the east Pacific, in depths ranging from around 100 m to more than 2800 m [2]. Geryonid crabs are non-swimming. The species demonstrates sexual dimorphism and the males are larger than the females [3]. Males and females of *C. maritae* show an affinity for different depths, tending males to be more prevalent at depths higher than 500 m and females to be more predominate in shallower water (400 to 500 m) [3,4,5]. Because of this distribution pattern, a depth restriction of 400 m was previously introduced aiming to protect female crabs [6]. Currently, in Angola, the protection of this species is ensured by setting seasons when the crab catching is interdicted. At the time it is allowed, the crab catch mainly occurs in the depths where females predominate. Because of this, females are more captured and consumed than males. No seasonal fluctuation has been observed in the reproductive cycle of *C. maritae*, and, therefore, this crab appears to fit typical year-round deep-sea reproductive pattern [4]. Red crabs grow slowly and are long-lived [7]. Specimens of *C. maritae* likely older than 25 years (carapace width of 165 mm) have already been observed off the Namibia coast [8].

*C. maritae* are found off the West African coast from Western Sahara to South West Africa and has been commercially fished in several countries, particularly in the Ivory Coast, Namibia, and Angola [5].

In the province of Namibe (Angola), *Chaceon maritae* is one of the main seafood resources, both for domestic consumption and export, mainly to Europe and Asia, which is an important commercial crab species [9]. In the last five years (2013 to 2017), the total catch of this species in Namibe was on average 1628.2 tons/year, which is the lowest value of 975 tons in 2014 and the highest 2586 tons in 2015 [10]. Generally, after catching, crabs are sent to seafood processing industries, where they are cooked (boiled) and marketed as a whole, body halves, or butchered and marketed in different products such as leg, claw, or flakes.

Several reports claimed that especially muscle and ovaries of different crab species, from different geographical origins and caught in different seasons, have a high nutritional value and can promote human health [11,12,13,14,15,16,17]. The nutritional benefits associated with sea food products, including marine crabs, are largely associated with their essential amino acids and essential fatty acids content [15], especially their content in long-chain ω3-polyunsaturated fatty acids (ω3-PUFA), as well as with their content in essential elements (like iron, zinc, selenium, or iodine) and vitamins and their low content in saturated fatty acids [13]. Long-chain ω3-PUFA seems to have beneficial effects for preventing cardiovascular diseases [18]. The precise mechanisms underlying ω3-PUFA cardioprotective effects are not fully understood and may include membrane modification, attenuation of ion channels, and regulation of pro-inflammatory gene expression and production of lipid mediators [19]. Furthermore, the consumption of ω3-PUFA also appears to have beneficial effects on ocular health [20] and in terms of the prevention or treatment of arthritis and other inflammatory and autoimmune disorders [21] as well as certain types of cancer [22]. Moreover, low dietary consumption of ω3-PUFA has been implicated in neuropsychiatric diseases, encompassing specific domains including development, neurodegeneration, cognition, or neuroinflammation [23].

Regardless of all the mentioned nutritional benefits, other studies pointed out that crab consumption could also pose some risks due to the presence of high levels of toxic elements such as cadmium [13,15,17], arsenic [17], chromium, and lead [7]. Their slow growth and close association with the benthos make them particularly susceptible to contamination with heavy metals and other toxins present in sediments and in the water column [7].

Despite being highly appreciated and consumed, the nutritional value of *C. maritae* from Namibe (Angola) has never been studied. Thus, the present work aimed to characterize the chemical composition of the edible tissues (muscle, ovaries, and hepatopancreas) of *C. maritae* caught in Namibe waters in two distinct seasons, in order to evaluate the risks and the benefits of their consumption. To reach this goal, the crab tissues were analyzed in terms of proximate chemical composition (fat, ash, protein, and humidity), fatty acid and amino acid profiles, cholesterol, essential minerals (macro and trace), and toxic elements.

## 2. Materials and Methods

### 2.1. Biological Material

A total of 56 female *Chaceon maritae* were collected off the southern coast of Angola, 7 to 14 miles south of Namibe province, at a depth of 200 to 400 m and into two different seasons: In March 2015, during the hot and rainy season (*n* = 26), and in October 2015, immediately after the colder and dry season (*n* = 30). In 2015, the superficial sea temperature ranged between 21 and 28 °C in March and between 16 and 27 °C in October [24]. Specimens were caught in traps, transported to the industrial facility, and then washed with fresh water and cooked (water boiled) in seawater (approximately 15 min, 100 °C) with sodium chloride (2 g/L) and the chili pepper *Capsicum chinense* (175 mg/L). After cooking, crabs were cooled to room temperature, frozen at −35 °C (freezer tunnel), and stored at −18 °C for two days. Frozen crabs were then suitably packaged in isothermal boxes and shipped by air to Portugal.

Once in the laboratory, crabs were measured (carapace width and length), since the body carapace length/width relationship is important for the purpose of its commercial exploitation [25], weighted, and subjected to chemical analyses. The edible tissues of each crab were separated in muscle, ovaries, and hepatopancreas and, subsequently, each tissue was homogenized with a grinder (Retsch Grindomix GM200, Retsch, Haan, Germany, 5000 rpm) until the complete visual disruption of the tissue (material: polypropylene cup and stainless-steel knives) and stored at –80 °C until further analyses. A portion of the sample was freeze-dried at –40 °C and low pressure (approximately 10^−1^ atm, Heto PowerDry LL3000 Freeze Dryer, Thermo Fisher Scientific, Karlsruhe, Germany) for 48 h and stored at –80 °C until further analyses.

### 2.2. Proximate Chemical Composition and Energy Content

Moisture, ash, and total fat were determined according to the Association of Official Analytical Chemists methods [26]. Moisture was determined by drying the sample overnight at 105 °C (Laboratory heater, Memmert UL500, Schawabach, Germany). Ash was obtained after dried sample combustion for 16 h at 500 °C (Muffle furnace, Heraeus Hanau MR170E, Hanau, Germany) and total fat content was determined with the Soxhlet extraction method using ethyl ether (40–60 °C, 7 h). Crude protein was analyzed using the Dumas method, in a nitrogen analyzer (LECO FP-528) calibrated with EDTA, using a conversion factor of 6.25 [27]. The results were expressed in g per 100 g wet weight. The energy content was estimated as: proteins, 4.27 kcal g^−1^ wet weight, lipids, 9.02 kcal g^−1^ wet weight, and 1 kcal = 4.184 kJ [28].

### 2.3. Fatty Acid Analysis

Fatty acid methyl esters (FAME) were prepared by acid-catalyzed transesterification according to the procedure of Lepage & Roy [29], modified by Cohen et al. [30]. Furthermore, 50 μL of an internal standard solution (C21:0) (concentration = 10 mg/mL) and 5 mL of acetyl chloride/methanol (1:19) were added to each sample. Samples were injected into a gas chromatograph equipped with an auto-sampler (Scion 456 GC, Livingston, UK). The separation of FAME was carried out with helium as carrier gas in a DBwax-polyethylene glycol capillary column (30 m length × 0.25 mm internal diameter, 0.25 μm thickness) programmed at 180 °C for 5 min, raised to 220 °C at 4 °C min-1, and maintained at 220 °C for 25 min. Detection of FAME was done on a flame ionization detector at 250 °C. The identification and quantification of FAME were accomplished through calibration curves using Sigma standards (Sigma-Aldrich Corp., St. Louis, MI, USA): Supelco PUFA No.1 (Marine Source, 99%—Ref. 47033) and PUFA No.3 (Menhaden oil 99%—Ref. 47085-U). Results were expressed in mg/100 g of the edible part on fresh weight basis. To evaluate the tendency of cooked crab to influence the incidence of coronary heart disease, the atherogenic (AI) and thrombogenic (TI) indices were calculated, according to the following formulas [31]: AI = (12:0 + 4 × 14:0 + 16:0)/(ω6 PUFA + ω3 PUFA + MUFA), TI = (14:0 + 16:0 + 18:0)/(0.5 × MUFA + 0.5 × ω6PUFA + 3 × ω3PUFA + ω3PUFA/ω6 PUFA).

### 2.4. Cholesterol Determination

A colorimetric/enzymatic method was used to determine cholesterol in foodstuffs and other materials (Cat. No.10139050035, R-Biopharm, Darmstadt, Germany). The extraction of free cholesterol was conducted as follows: 1 g of sample was extracted with 10 mL of isopropanol for 1 h at 40 °C and shaken every 10 min. After the extraction, the undissolved components were removed by filtration using the Whatman Nº42 filter. The absorbance of sample blank and samples were read against air at 405 nm. The results were expressed as mg of free cholesterol/100 g of the edible portion on fresh weight basis. Due to limitations in the sample amount, this analysis was only performed in muscle.

### 2.5. Amino Acid Analysis

The amino acid analysis was performed according to the procedure described by Mota et al. [32], using the Waters AccQ Fluor reagent kit, containing 6-aminoquinolyl-N-hydroxysuccinimidyl carbamate for derivatization, AccQ Tag Ultra Eluent A (ammonium formate in water/acetonitrile/formic acid = 84:10:6) diluted in 95% of deionized water and AccQ Tag Ultra Eluent B (2% formic acid in acetonitrile) for mobile phase, which were all obtained from Waters Corporation Company (Milford, MA, USA). The test portions (20 mg) were weighted into proper quartz digestion vials and one millilitre of hydrochloric acid (6 N), containing 0.5 % phenol, and 200 µL of internal standard (25 mM of D-Norvaline) were added. Samples were then digested using a closed-vessel microwave digestion system (Milestone ETHOS 1 Series, Sorisole, Italy), in anaerobic conditions using nitrogen gas purges and a vacuum pump, at 160 °C (15 min to increase the temperature to 160 °C, 10 min at 160 °C, and 90 min to cool). After complete hydrolysis, the extracts were neutralized with sodium hydroxide (6 N), filled up to a total volume of 10 mL with deionized water, filtered (common filter paper) and subjected to derivatization by adding 10 µL of sample, 80 µL of buffer, and 20 µL of reconstituted derivatization reagent, and heated to 55 °C for 10 min.

The chromatographic determination was performed in an Acquity UPLC system from Waters (Milford, MA, USA), equipped with photodiode array (PDA) detector, and a BEHC18 column (100 mm × 2.1 mm i.d., 1.7_m; Waters). The eluents used were AccQTagultra diluted in 95% of deionized water (eluent A) and AccQTag ultra (eluent B). The following gradient conditions used were: 0–0.54 min, 99.9% A–0.1% B, 5.74 min, 90. 9% A–9.1% B, 7.74 min, 78.8% A–21.2% B, 8.04 min, 40.4% A–59.6% B, 8.70–10 min, 99.9%, and A–0.1% B. This was conducted within 10 min of gradient chromatographic run time. The flow rate was 0.7 mL/min, the column temperature was kept at 55 °C, the injection volume was 1 µL, and the detection wavelength was set at 260 nm. Derivatized amino acids were identified and quantified by comparison with the retention times and areas ratios of the standard amino acid mixture with the internal standard. Results were expressed in mg per 100 g of edible portion on fresh weight basis and in mg/g protein.

Essential amino acid scores (AS) were calculated with respect to reference amino acid requirements for adults: isoleucine (30 mg g^−1^ protein), leucine (59 mg g^−1^ protein), lysine (45 mg g^−1^ protein), methionine (16 mg g^−1^ protein), phenylalanine + tyrosine (38 mg g^−1^ protein), threonine (23 mg g^−1^ protein), valine (39 mg g^−1^ protein), and histidine (15 mg g^−1^ protein) [33]. Results of AS were achieved by comparing the content of each essential amino acid in the protein/diet with its requirements, according to the equation: AS (%) = (mg of amino acid per g of protein/mg of amino acid requirement) × 100. The ratio of essential amino acids to non-essential amino acids was also calculated. Due to limitations in the sample amount, this analysis was only performed in the muscle.

### 2.6. Minerals

Magnesium (Mg), K, Na, and Fe were determined by flame atomic absorption spectrophotometry (Spectr AA 55B spectrophotometer, Varian, Palo Alto, CA, USA) with a background deuterium correction, according the procedure described by Jorhem [34]. Calibration curves of, at least, five different concentrations were used to quantify each element. Mercury (Hg) was measured with a Mercury Analyser spectrophotometer (AMA 254, Leco, St. Joseph, Michigan, USA) by thermal decomposition, amalgamation, and atomic absorption spectrometry [35]. Phosphorus (P) was determined by molecular absorption spectrophotometry at 430 nm Molecular absorption spectrophotometry at 430 nm (UNICAM UV/Vis UV2, Thermo Scientific, Karlsruhe, Germany) after incineration followed by acid digestion and colorimetric measurement of a yellow compound resulting from the reaction between phosphorus and an ammonium vanadate and ammonium molybdate mixture [36]. Accuracy of Mg, K, Na, Fe, Hg, and P determination methods was checked through the analysis of certified reference material (DORM-4 - fish protein). Vanadium (V), Cr, Co, Mn, Ni, Cu, Zn, As, Cd, and Pb concentrations were analyzed in freeze dried, ground, and homogenized samples after digestion with HNO_3_ (bi-distilled, 65 % *v/v*) and H_2_O_2_ (sp, 30 % *v/v*), as described in Raimundo et al. [37]. Procedural blanks were prepared using the same analytical procedure and reagents. Blanks accounted for less than 1% of the total metal in samples. Concentrations were determined using an X-Serie ICP-MS (Thermo Scientific, Manchester, UK), equipped with a Peltier impact bead spray chamber and a concentric Meinhard nebulizer. A 9-point calibration curve was used to quantify each element, using a commercial solution of indium (In) as an internal standard (Merck, Darmstadt, Germany, CertiPUR^®^). Analytical methods accuracy was assessed by the analysis of international certificate reference material (DORM-2—dogfish muscle, DORM-3—fish protein). No significant (*p* > 0.05, Mann-Whitney) differences were observed between obtained results and certified values.

Iodine (I) and Se contents were determined by Inductively Coupled Plasma Mass Spectrometry (ICP-MS). Iodine was determined after alkaline digestion in a graphite block system, according to ES EN 15111:2007 [38] and selenium after acidic microwave pressure digestion, according to EN 15763:2009 [39]. All reagents used were of high analytical grade. Working standard solutions of iodine were prepared from single-element high purity ICP stock standard containing 1000 mg/L of iodine (Inorganic Ventures, Christiansburg, Virginia). Internal standard solutions of rhodium (10 mg/L) and tellurium solution (1000 mg/L) were purchased from Inorganic Ventures (Christiansburg, VA, USA) and Merck (Darmstadt, Germany), respectively. Working standard solutions of selenium were prepared from single element high purity ICP stock standard containing 1000 mg/L of selenium (SCP Science, Marktoberdorf, Germany). Internal standards of germanium, indium, and yttrium solutions (1000 mg/L) were purchased from Inorganic Ventures (Christiansburg, VA, USA). The results were obtained in triplicate analytical samples under conditions of quality assurance supported by the requirements described in NP EN ISO/IEC 17025:2005 [40]. In this study, precision and accuracy, limit of quantification (LoQ), selectivity, and an effective internal and external quality control program [Certified Reference Materials (CRM), spiked samples with chemical standards and participation in adequate PT Schemes] were carried out to ensure the analytical quality. Iodine and Se were quantified using a calibration curve with correlation coefficients ≥ 0.9995. For each element, an internal quality control were used with an acceptance criterion of 10%.

Results were expressed in mg per 100 g of the edible portion on fresh weight basis. Due to limitations in the sample amount, this analysis was not performed in all tissues.

### 2.7. Statistics

One-way or factorial analysis of variance (ANOVA) followed by the Tukey’s test was used to identify significant differences between tissues and/or seasons in biometric data, proximate chemical composition, cholesterol, fatty acid, and amino acid profiles as well as essential and toxic elements. Whenever the assumption of normality and variance homogeneity (Cochran, Hartley, and Bartlett tests) were not verified, nonparametric analysis of variance (Kruskall-Wallis) and multiple comparison tests were applied. All statistical analyses were tested at the 0.05 level of probability with the software STATISTICA™ 7.0 (StatSoft, Tulsa Oklahoma, OK, USA).

## 3. Results and Discussion

### 3.1. Biometric Data and Proximate Chemical Composition

The biometric data of *Chaceon maritae* (Table 1) did not show significant differences between seasons either in terms of carapace length (CL) or in terms of weight. On the contrary, specimens collected in March presented a carapace width (CW) significantly higher than specimens collected in October. *C. maritae* CW sizes up to 165 mm were reported for males and up to 120 mm for females, although relatively few female crabs grow larger than 100 mm (CW) [5,8]. The differences in CW observed between seasons suggest that crabs caught in March were older than crabs caught in October. However, in both seasons, all crabs analyzed were roe-bearing females.

The analysis of the chemical composition and nutritional value of *C. maritae* was carried out on previously water boiled and frozen crabs, since this is the main form as red crabs from Namibe are marketed. When analyzing the effect of boiling on the proximate chemical composition of several crab species, other authors reported controversial results. Thus, when some reported no significant differences between raw and cooked tissues [41], others reported lower moisture and higher ash and protein content after cooking [15,16]. However, even when there were variations in moisture, protein, and ash contents of raw and cooked tissues, these variations occurred within narrow ranges of values [15,16].

The proximate chemical composition of the edible tissues of boiled *C. maritae* caught during March and October is presented in Table 2. Although there may have been some change in relation to raw meat, the muscle of boiled *C. maritae* collected off the coast of Namibe presented a proximate chemical composition in line with that of other raw or cooked geryonid crab species. Thus, *C. maritae* from Namibe presented a protein, ash, and fat content similar to those reported for raw *C. affinis* from Madeira (Portugal) (17.8; 2.3 and 0.7 g/100 g, for protein, ash, and fat, respectively) [11], to steamed *C. quinquedens* from Nova Scotia (Canada) (15.1, 1.75, 0.88 g/100g for protein, ash, and fat, respectively) [42] and a fat content similar to that reported to boiled *C. quinquedens* from New Jersey (United States of America) (0.9 g/100 g) [43]. Moreover, the proximate chemical composition was also similar to adult females from other crab species like swimming crab (*Portunus trituberculatus*) (15.73 and 1.20 g/100g of protein and fat, respectively) [17] or blue swimmer crab (*Portunus pelagicus*) (18.4 and 1.08 g/100g of protein and fat, respectively) [14].

In general, significant differences were observed between the chemical composition of the different tissues (Table 2). Thus, the muscle has the highest moisture and ash content, the ovaries have the highest protein content, and the hepatopancreas has the highest fat content. It was observed that muscle and ovaries contained more protein than hepatopancreas and that hepatopancreas and ovaries were richer in fat. Due to their higher fat content, ovaries and hepatopancreas presented an energy value higher than the muscle. Other authors reported similar patterns of tissue composition in other species of crabs from other locations, like wild-caught and pond-reared swimming crab *(Portunus trituberculatus*) adult females [17], blue swimmer crab (*Portunus pelagicus*) [14], Atlantic spider crab (*Maja brachydactyla*) [13], or brown crab (*Cancer pagurus*) [12]. However, in some species, lower fat contents in muscle, ovary, and hepatopancreas has been reported [12,13]. The differences observed between tissues could be related to their functions. The muscle is a structural tissue mostly composed of proteins and structural lipids, whereas hepatopancreas and ovaries are rich in storage lipids necessary to fulfil crabs’ physiological needs [15].

When comparing the proximate chemical composition in the two seasons under analysis (March and October), the most pronounced difference observed was in the hepatopancreas moisture and fat contents. Thus, hepatopancreas of animals caught in October presented a moisture content lower and a fat content higher (approximately 16%) than hepatopancreas of animals caught in March. These differences could be related to environmental variations, like changes in temperature or in nutrients availability, as well as differences in animal physiology [15]. Seasonal fluctuations in fat content were also reported in brown meat (mostly gonads plus hepatopancreas) of *Cancer pagurus* from the Scottish coast, whose fat content, either raw or boiled, was higher in crabs caught during the summer than in crabs caught during the spring [15].

### 3.2. Fatty Acid Profile and Cholesterol Content

The fatty acid profile of edible tissues of *C. maritae* caught in March and in October is presented in Table 3. Significant differences (*p* < 0.05) were observed both among tissues and between seasons. In both seasons, polyunsaturated fatty acids (PUFA) were the main group of fatty acids in muscle, which was followed by monounsaturated fatty acids (MUFA) and, lastly, by saturated fatty acids (SFA). On the other hand, MUFA were the main group in hepatopancreas, which was followed by SFA in October or by PUFA in March. In March, PUFA and MUFA (*p* > 0.05) were the main groups of fatty acids in ovaries, but, in October, PUFA content was significantly higher than MUFA content. In ovaries, SFA were the minor group of fatty acids in both seasons.

In all tissues and in both seasons, palmitic acid (C16:0) was the main SFA, accounting for about 50% of the total SFA. The second most important SFA was stearic acid (C18:0) accounting for about 20% in muscle and hepatopancreas and about 15% in ovaries. Within MUFA, the oleic acid (C18:1ω9) was the most abundant. As a matter of fact, oleic acid accounted for more than 50% of the total in all tissues and in both seasons analyzed. In muscle and ovaries, oleic acid was followed by the mixture of palmitoleic acid (C16:1ω7) and its isomer (C16:1ω9), whereas, in the hepatopancreas, it was followed by eicosenoic acids (C20:1ω9 + ω7). Eicosapentaenoic acid (EPA) (C20:5ω3) and docosahexaenoic acid (DHA) (C22:6ω3) were the main PUFA in all tissues and in both seasons analyzed. Despite some variations within the percentage of each fatty acid, the fatty acid profile of cooked muscle from *C. maritae* from Namibe was in accordance to those reported for the muscle of other cooked [16,43] or raw [12,13,14,16,17] crab species. The effect of boiling on the fatty acid profile seems to be controversial. Thus, while Maulvault et al. [15] showed that boiling strongly decrease SFA, MUFA and PUFA contents both in muscle and brown meat *of Cancer pagurus*, Risso and Carelli [16] showed that boiling leads to an increase of PUFA content of *Lithodes santolla*.

Similar to what was observed in red crab from Namibe, MUFA were also the main group of fatty acids in the hepatopancreas of males and females of *Cancer pagurus* from English Channel and the Scottish coast [12]. However, in other species, namely in *Maja brachydactyla* [13] and *Portunus pelagicus* [14], SFA were the main group of fatty acids in hepatopancreas.

The main differences observed among tissues were in the MUFA C20:1(ω11 + ω9 + ω7) and C22:1ω11, which presented higher values in hepatopancreas than in ovaries and muscle, and in DHA and EPA, where concentrations in hepatopancreas, especially in October, were lower than in muscle and in ovaries. The differences observed between tissues must be related to their different physiological functions. The higher percentage of C20:1(ω11 + ω9 + ω7) in hepatopancreas was also reported by others [12,13,14]. The presence of cetoleic acid (C22:1ω11) was also reported in the cooked muscle of *Chaceon quinquedens* (1.7 %) and *Cancer irroratus* (4.3 %) from Northwest Atlantic [43].

When comparing the fatty acid profile in both seasons, it was possible to observe that, in October, the percentage of SFA and MUFA in muscle and hepatopancreas increased and the percentage of PUFA decreased, while, in ovaries, the values were constant. As previously mentioned, these differences could be a consequence of changes in temperature, nutrient availability, or animal physiology. According to Cuculescu et al. [44], fatty acids may play an important role in the adjustments of membrane fluidity to variations in environmental temperature. These authors suggested that the accumulation of long-chain PUFA before the cold season could make crabs more able to adapt to rapid decreases in temperature and the accumulation of saturated fatty acids before the hot season more capable of adapting to rapid increases in temperature [44]. Thus, the higher percentage of SFA and MUFA in muscle and hepatopancreas observed in October (immediately after the cold season) and the higher percentage of PUFA observed in March (before the cold season) could be related to this temperature adaptation.

Together, DHA and EPA accounted for about 31% to 35% of total fatty acids in muscle, 23% to 25% in ovaries, and 13% to 19% in hepatopancreas. The content of these two important ω3-fatty acids in the edible crab tissues was higher than 200 mg/100 g in the muscle. This intake is adequate for the primary prevention of cardiovascular disease based on European Food Safety Authority (EFSA) recommendation intake for adults (250 mg/day) [45]. Moreover, the ratio ω3-PUFA/ω6-PUFA ranged from 3.04 (hepatopancreas in October) to 5.70 (muscle in March), the atherogenic index from 0.24 (muscle in March) and 0.45 (hepatopancreas in October), and the thrombogenic index from 0.13 (muscle in March) to 0.30 (hepatopancreas in October). All these indices are important to evaluate the nutritional quality of fat present in food. An imbalanced ω3/ω6 ratio shifts the physiological state to one that is proinflammatory, prothrombotic, and pro-aggregatory, with increases in blood viscosity, vasospasm, vasoconstriction, and cell proliferation. Western diets are characterized by high ω6 and low ω3 intake, which leads to a ω6/ω3 around 20:1, whereas a 1:1 to 2:1 ω6-PUFA to ω3-PUFA should be the target ratio for health [46]. The atherogenic and thrombogenic indices were created by Ulbricht & Southgate [31] to evaluate the predisposition of food to influence the incidence of coronary heart disease, which has the potential to promote thrombus formation and increased plasma cholesterol levels.

The high ω3-PUFA/ ω6-PUFA are in accordance with the values reported from other species such as *M. brachydactyla* [13], *P. trituberculatus* [17], or *C. pagurus* [12] and higher than the values reported to *P. pelagicus* [14]. The AI and TI indices are similar to that reported to *C. pagurus* [12] and to the muscle and gonads of *M. brachydactyla* [13], and lower than those reported in other food items like lamb chop (IA 1.00; IT 1.33), raw minced beef (IA 0.72; IT 1.27), or chicken (IA 0.5; IT 0.95) [30]. According to these three indices, the edible tissues of *C. maritae* had high nutritional quality.

Muscle cholesterol levels were similar in both seasons (Table 3). The values obtained were like that reported among other species, such as cooked *Chaceon quinquedens*, *Cancer borealis*, and *Cancer irroratus* [43], but higher than values reported among raw or cooked *Lithodes santolla* [16] and raw *C. pagurus* [12] and *M. brachydactyla* [13]. These differences could be related to differences between species and/or environmental conditions or with the culinary processes that the crabs suffered before being analyzed. Risso & Carelli [16] reported significant differences in cholesterol levels between raw (37.3 mg/100 g) and cooked (51.0 mg/100g) *Lithodes santolla*, which could not be explained by only differences in moisture content. The authors suggested that these differences could be related to alterations occurring in protein-cholesterol complexes of tissues after heating. Cholesterol plays a role in the membrane’s fluidity and permeability and is a precursor of vitamin D, adrenal and sex steroid hormones, and bile salts. Most authoritative bodies do not provide a maximum amount for cholesterol consumption, but, when they do, the advice is to not exceed 300 mg/day [47]. Thus, although having a cholesterol content higher than that found in several other crab species, the cholesterol content of *C. maritae* from the Namibe was still significantly lower than this maximum.

### 3.3. Amino Acid Profile

Proteins are essential elements of a healthy diet, which allow both growth and maintenance of the 25,000 proteins encoded within the human genome, as well as other nitrogenous compounds [33]. The amino acid profile is one of the factors that influence dietary protein quality. Amino acid could be classified as nonessential amino acids (NEAA), such as those that can be synthesized by the body, or essential amino acids (EAA), which are those that the human body cannot synthesize and, therefore, must be provided through diet. According to the World Health Organization [33], histidine, isoleucine, leucine, lysine, methionine, phenylalanine, threonine, tryptophan, and valine are EAA, whereas aspartic and glutamic acids, asparagine, alanine, serine, cysteine, tyrosine, glycine, arginine, glutamine, and proline are NEAA. However, some NEAA, such as cysteine, tyrosine, glycine, arginine, glutamine, or proline, are termed conditionally indispensable since they became essential under specific pathological or physiological conditions [48].

The amino acid profile of *C. maritae* muscle is given in Table 4. Tryptophan, asparagine, and glutamine were destroyed by acid hydrolysis and were not determined. No significant differences were observed between the amino acid profiles of crabs caught in March or in October. Thus, in both seasons, glutamic acid was the main NEAA, which was followed by aspartic acid and arginine. In what concerns EAA, leucine and lysine were the ones that presented higher contents. In general, these results agree with those found in the literature for other crab species [12,13,14,17]. In both seasons, NEAA dominates the protein content resulting in EAA/NEAA ratios lower than 1. This result is also in agreement with the scientific literature since it is known that crustacean muscle contains more non-essential amino acids, which are synthesized or modified in the body, than essential amino acids [12].

The essential amino acid score (EAAS) was calculated concerning the reference amino acid pattern for adults (Table 5). Results showed that muscle protein is well balanced when concerning EAA, presenting EAAS higher than 100 for all EAA except for valine, which reached 84 and 89 in March and October, respectively. The limiting amino acids differ among muscle protein of different crab species. For example, isoleucine was reported as the limiting amino acids in *M. brachydactyla* [13] and methionine in *C. pagurus* [12], while tryptophan was reported as the limiting amino acid in *P. pelagicus* [14].

Results showed that *C. maritae* protein from Namibe has high quality and could be considered a good source of EAA. Intakes of EAA higher than those in the reference amino acid pattern could be useful to complement low-quality proteins from other dietary sources. Moreover, high EAA intakes could be important, not only to support growth or N-balance but also to support other functions such as lean body mass retention, cell signaling, bone health, glucose homeostasis, and satiety induction [48].

### 3.4. Essential Elements and Contaminants

Minerals are essential for human health, so their quantification is important for assessing the nutritional quality of foods [17]. Mineral concentration in the edible tissues of boiled *C. maritae* caught in March and October are presented in Table 6. In general, the mineral content of crabs caught in March or in October did not show marked differences. In muscle, Na was the most abundant macro element, which was followed by K, P, and Mg. The same trend has been reported to other crab species [13,17]. However, red crab from Namibe presented an Na concentration much higher than those of other species, which should be related to the addition of sodium chloride to the water used for cooking.

Significant differences were generally observed between trace metal content of the different tissues (Table 6), with these contents being lower in muscle than in ovaries and hepatopancreas. The differences in trace metal concentrations verified among tissues were also reported by other authors, and could be related to their distinct physiological functions [13]. In agreement with the results found in other crab species [13,17], Zn was the most abundant trace metal in all crab tissues. The concentration of this metal was particularly high in ovaries. The selective incorporation of Zn in the ovary tissue suggests that this metal could be important for *C. maritae* oogenesis. Other crustaceans also showed an accumulation of Zn in the ovary tissue, which suggests the incorporation of this metal into metalloenzymes and its involvement in the stabilization of storage proteins used during embryogenesis [52,53]. The Adequate Intake values of macro and trace metals set by EFSA (Table 6) indicate that *C. maritae* from Namibe is a particularly good source of Se, I, Cu, and Zn. From the nutritional point of view, the high Zn, I, and Se content are particularly important, since deficiencies in these minerals are widespread, including in European countries [54,55]. Deficiencies in these minerals can contribute to poor growth, intellectual impairments, perinatal complications, and increased risk of morbidity and mortality [54].

Arsenic, Hg, Cd, and Pb are the elements of greatest concern for seafood safety [56], with Cd being the one of greatest concern with regard to crab contamination. Previous studies have shown that considerable levels of Cd could be found in the hepatopancreas of crabs from distinct geographic locations [13,15,57]. For this reason, the European Union published an information note saying that consumption of brown crab meat could lead to unacceptable cadmium exposure and that consumers should be advised to avoid or limit its consumption [58]. Cadmium concentrations in the edible tissues from *C. maritae* from Namibe were very low and below the maximum levels laid down in European regulations (Table 6), which shows that its consumption presents no risk of exposure to high levels of Cd. Furthermore, results obtained showed that the concentrations of Pb and Hg in the edible tissues of *C. maritae* were also below the maximum levels laid down in European regulations (Table 6). At present, no maximum limit is set by the European Union for As in seafood. However, the values obtained were low when compared to other crab species caught in other locations [7,13,17,56,59]. Moreover, it is known that the predominant form of As in seafood is the non-toxic arsenobetaine [56]. The low concentration of toxic elements in *C. maritae* from Namibe could be related not only with species characteristics, but also with other factors that could influence the uptake of metals, like the concentration of exposure, climate, salinity, and possible synergistic and antagonistic effects of other metals and organic compounds that could be present [7,57]. Results obtained showed that *C. maritae* from Namibe is a safe food in what concerns heavy metal contamination.

## 4. Conclusions

Significant differences in the nutritional composition of *Chaceon maritae* from Namibe (Angola) were recorded according to tissue (muscle, ovary, and hepatopancreas) and to the caught season (March and October). Despite these differences, in both seasons, *C. maritae* muscle prove to be a good source of protein, essential amino acids, polyunsaturated fatty acids, especially EPA and DHA, and essential elements, which include Zn, Se, I, and Cu. The muscle cholesterol level was moderate, but significantly lower than the maximum recommended daily intake. Ovaries and hepatopancreas are also a good source of protein but were richer in fat, particularly when caught in October. However, while ovarian fat is rich in PUFA, hepatopancreas fat presented higher values of MUFA and SFA. Nevertheless, hepatopancreas fat was rich in EPA and DHA and presented a ω3/ω6 ratio higher than three in both seasons. Hepatopancreas and ovaries are also good sources of Cu and, especially in ovaries, of Zn. Moreover, in both seasons, all the edible tissues of *C. maritae* analyzed presented very low contents of toxic elements. The data suggest that cooked red crab from Namibe, particularly muscle and ovaries, is a nutritious and healthy food for human consumption.

## Figures and Tables

**Table 1:** Biometric data of *C. maritae* caught in March and October (average ± standard deviation, values between brackets are minimum and maximum values of each measurement).

	March	October
Number of specimens	26 (females)	30 (females)
Carapace width (CW) (mm)	87.1 ± 3.2 ^a^ (81-95)	79.6 3.1 ^b^ (72–89)
Carapace length (CL) (mm)	75.5 ± 3.4 ^a^ (69–84)	73.7 ± 3.8 ^a^ (67–85)
Total weight (g)	156.8 ± 29.5 ^a^ (99.0–211.0)	145.0 ± 20.7 ^a^ (110.0–182.0)

In each row, different letters denote significant differences (*p* < 0.05).

**Table 2:** Proximate composition (g/100 g wet weight) and energetic value (kcal/100 g) of the edible tissues of boiled *C. maritae* caught in March and October (average ± standard deviation).

Content	March			October		
	Muscle	Ovaries	Hepatopancreas	Muscle	Ovaries	Hepatopancreas
Moisture	76.6 ± 0.4 ^a^	60.2±0.4 ^e^	67.3 ± 0.1 ^c^	75.8 ± 0.1 ^b^	60.0 ± 0.1 ^e^	64.4 ± 0.1 ^d^
Ash	3.2 ± 0.1 ^a^	2.1 ± 0.1 ^d^	2.6 ± 0.1 ^b^	3.0 ± 0.1 ^a^	2.0 ± 0.0 ^d^	2.4 ± 0.1 ^c^
Protein	17.7 ± 0.4 ^b^	21.4 ± 0.4 ^a^	11.5 ± 0.4 ^c^	17.1 ± 0.1 ^b^	21.9 ± 0.2 ^a^	12.2 ± 0.2 ^c^
Fat	1.0 ± 0.0 ^e^	11.8 ± 0.2 ^d^	16.8 ± 0.1 ^b^	1.0 ± 0.0 ^e^	12.7 ± 0.1 ^c^	20.0 ± 0.3 ^a^
Energy	84.5 ± 2.4 ^d^	196.9 ± 3.7 ^c^	200.2 ± 0.4 ^c^	82.2 ± 0.3 ^d^	207.9 ± 0.5 ^b^	232.3 ± 3.0 ^a^

In each row, different letters denote significant differences (*p* < 0.05).

**Table 3:** Fatty acid and cholesterol composition of boiled *C. maritae* caught in March and October (average ± standard deviation).

Fatty acid	March			October		
	Muscle	Ovaries	Hepatopancreas	Muscle	Ovaries	Hepatopancreas
Saturated						
14:0 (%)	1.71 ± 0.15 ^a^	3.60 ± 1.86 ^a^	3.40 ± 0.40 ^a^	2.05 ± 0.28 ^a^	2.27 ± 0.06 ^a^	3.93 ± 0.69 ^a^
16:0 (%)	10.95 ± 0.50 ^a^	13.59 ± 2.86 ^a^	12.54 ± 0.18 ^a^	12.03 ± 0.87 ^a^	11.41 ± 0.12 ^a^	13.61 ± 0.42 ^a^
18:0 (%)	4.94 ± 0.06 ^b^	3.24 ± 0.18 ^c^	4.85 ± 0.16 ^b^	5.17 ± 0.02 ^a,b^	3.52 ± 0.01 ^c^	5.47 ± 0.08 ^a^
Other SFA (%)	3.49 ± 0.09 ^b^	4.22 ± 0.54 ^a^	4.09 ± 0.12 ^a,b^	3.54 ± 0.08 ^a,b^	3.72 ± 0.06 ^a,b^	4.48 ± 0.22 ^a^
ΣSFA (%)	21.07 ± 0.62 ^a^	24.65 ± 5.07 ^a^	24.87 ± 0.58 ^a^	22.78 ± 1.21 ^a^	20.90 ± 0.11 ^a^	27.48 ± 1.25 ^a^
ΣSFA (mg/100 g ww)	143.9 ± 4.2 ^d,C^	2695 ± 516 ^c,B^	3927 ± 107 ^b,C^	155.6 ± 10.4 ^d,C^	2480 ± 37 ^c,C^	5178 ± 284 ^a,B^
Monounsaturated						
16:1(ω9 + ω7) (%)	4.50 ± 0.22 ^b^	8.42 ± 2.07 ^a^	5.78 ± 0.15 ^b^	5.18 ± 0.43 ^b^	6.87 ± 0.07 ^a,b^	6.17 ± 0.27 ^a,b^
18:1(ω9) (%)	12.77 ± 0.45 ^b^	14.88 ± 0.55 ^a^	15.27 ± 0.99 ^a^	14.27 ± 0.07 ^a,b^	15.66 ± 0.01 ^a^	16.08 ± 0.57 ^a^
18:1(ω7) (%)	4.23 ± 0.06 ^cd^	4.06 ± 0.22 ^d^	5.23 ± 0.07 ^b^	4.56 ± 0.03 ^c^	4.41 ± 0.00 ^c,d^	5.70 ± 0.04 ^a^
20:1(ω11 + ω9 + ω7) (%)	3.40 ± 0.29 ^d^	3.66 ± 0.59 ^d^	6.19 ± 0.11 ^b^	3.94 ± 0.34 ^c,d^	5.01 ± 0.04 ^c^	7.76 ± 0.07 ^a^
22:1(ω11) (%)	1.67 ± 0.22 ^c,d^	1.42 ± 0.24 ^d^	3.15 ± 0.10 ^b^	1.74 ± 0.18 ^c,d^	2.14 ± 0.01 ^c^	4.27 ± 0.09 ^a^
Other MUFA (%)	2.11 ± 0.05 ^b^	1.46 ± 0.36 ^c^	1.95 ± 0.06 ^b^	2.17 ± 0.04 ^b^	2.30 ± 0.01 ^b^	2.97 ± 0.10 ^a^
Σ MUFA (%)	28.68 ± 0.92 ^e^	33.90 ± 1.28 ^c,d^	37.56 ± 1.03 ^b^	31.86 ± 0.23 ^d^	36.38 ± 0.01 ^b,c^	42.95 ± 0.33 ^a^
Σ MUFA (mg/100 g ww)	195.9 ± 6.3 ^e,B^	3714 ± 99 ^d,A^	5928 ± 126 ^b,A^	217.6 ± 8.6 ^e,B^	4318 ± 20 ^c,B^	8094 ± 303 ^a,A^
Polyunsaturated						
18:2(ω6) (%)	0.78 ± 0.08 ^b,c^	0.85 ± 0.02 ^b,c^	1.06 ± 0.06 ^a^	0.77 ± 0.01 ^c^	0.81 ± 0.01 ^b,c^	0.94 ± 0.02 ^a,b^
18:3(ω3) (%)	0.21 ± 0.05 ^c^	0.31 ± 0.03 ^b^	0.41 ± 0.01 ^a^	0.19 ± 0.01 ^c^	0.30 ± 0.00^b^	0.34 ± 0.01 ^a,b^
20:4(ω6) (%)	3.70 ± 0.05 ^a^	2.98 ± 0.35 ^b,c^	2.76 ± 0.07 ^c^	3.43 ± 0.08 ^a,b^	3.12 ± 0.01 ^b,c^	2.06 ± 0.02 ^d^
20:5(ω3) (%)	17.29 ± 0.49 ^a^	11.54 ± 1.66 ^b^	8.21 ± 0.22 ^c^	15.91 ± 0.43 ^a^	11.92 ± 0.09 ^b^	5.75 ± 0.02 ^c^
22:5(ω3) (%)	2.18 ± 0.03 ^a,b^	2.54 ± 0.68 ^a,b^	2.17 ± 0.11 ^a,b^	1.90 ± 0.09 ^a,b^	2.99 ± 0.03 ^a^	1.70 ± 0.04 ^b^
22:6(ω3) (%)	17.33 ± 0.62 ^a^	11.50 ± 3.08 ^b,c^	11.14 ± 0.62 ^b,c^	14.98 ± 0.82 ^a,b^	12.72 ± 0.23 ^a,b^	6.68 ± 0.15 ^c^
Other PUFA (%)	5.42 ± 0.06 ^c^	6.32 ± 0.19 ^a^	6.22 ± 0.14 ^a,b^	5.11 ± 0.10 ^c^	6.49 ± 0.07 ^a^	5.94 ± 0.02 ^b^
Σ PUFA (%)	46.90 ± 0.97 ^a^	36.04 ± 5.95 ^b,c^	31.95 ± 0.96 ^c,d^	42.27 ± 1.51 ^a,b^	38.34 ± 0.03 ^a,b,c^	23.56 ± 0.13 ^d^
Σ PUFA (mg/100 g ww)	320.3 ± 6.6 ^c,A^	3957 ± 709 ^b,A^	5045 ± 186 ^a,B^	288.7 ± 12.2 ^c,A^	4550 ± 59 ^a,b,A^	4440 ± 84 ^a,b,C^
Σ PUFAω3 (%)	38.97 ± 0.98 ^a^	28.45 ± 5.67 ^b,c^	24.31 ± 0.92 ^c,d^	34.83 ± 1.37 ^a,b^	30.52 ± 0.04 ^a,b,c^	16.52 ± 0.16 ^d^
Σ PUFAω3 (mg/100 g ww)	266.2 ± 6.7 ^b^	3124 ± 666 ^a^	3839 ± 172 ^a^	237.9 ± 10.7 ^b^	3622 ± 50 ^a^	3114 ± 53 ^a^
Σ PUFAω6 (%)	6.83 ± 0.10 ^a^	5.91 ± 0.72 ^b^	6.46 ± 0.06 ^a,b^	6.18 ± 0.13 ^a,b^	6.25 ± 0.01 ^a,b^	5.44 ± 0.02 ^b^
Σ PUFAω6 (mg/100 g ww)	46.67 ± 0.65 ^c^	648 ± 88 ^b^	1020 ± 13 ^a^	42.21 ± 1.35 ^c^	742 ± 7 ^b^	1024 ± 23 ^a^
Σ PUFAω3/Σ PUFAω6	5.70 ± 0.16 ^a^	4.78 ± 0.40 ^c^	3.76 ± 0.14 ^d^	5.63 ± 0.11 ^a,b^	4.88 ± 0.01 ^b,c^	3.04 ± 0.02 ^e^
EPA + DHA (%)	34.62 ± 1.02 ^a^	23.04 ± 4.73 ^c^	19.35 ± 0.76 ^c,d^	30.89 ± 1.25 ^a,b^	24.64 ± 0.13 ^b,c^	12.60 ± 0.13 ^d^
EPA + DHA (mg/100 g ww)	236.4 ± 6.9 ^b^	2530 ± 555 ^a^	3056 ± 141 ^a^	210.9 ± 8.6 ^b^	2924 ± 38 ^a^	2375 ± 32 ^a^
SFA + MUFA + PUFA (%)	96.66 ± 0.39 ^a^	94.58 ± 0.51 ^b^	94.38 ± 0.39 ^b^	96.90 ± 0.54 ^a^	95.62 ± 0.16 ^a,b^	93.98 ± 1.05 ^b^
SFA + MUFA + PUFA(mg/100 g ww)	660.2 ± 2.69 ^e^	10366 ± 130 ^d^	14900 ± 155 ^b^	661.8 ± 31.1 ^e^	11348 ± 116 ^c^	17712 ± 670 ^a^
Atherogenic index	0.24 ± 0.02 ^a^	0.42 ± 0.18 ^a^	0.38 ± 0.03 ^a^	0.28 ± 0.03 ^a^	0.28 ± 0.00 ^a^	0.45 ± 0.05 ^a^
Thrombogenic Index	0.13 ± 0.00 ^b^	0.19 ± 0.07 ^a,b^	0.21 ± 0.01 ^a,b^	0.15 ± 0.01 ^b^	0.15 ± 0.00 ^b^	0.30 ± 0.01 ^a^
Cholesterol (mg/100 g)	62.8 ± 2.2 ^a^	na	na	79.2 ± 9.3 ^a^	na	na

Only fatty acids that account for more than 1% of the total are listed, (ww) wet weigh, (SFA) Saturated fatty acids; (MUFA) Monounsaturated fatty acids, (PUFA) Polyunsaturated fatty acids, (EPA) Eicosapentaenoic acid, (DHA) Docosahexaenoic acid, (na) not analyzed. In each row, different lower-case superscript letters denote significant differences among samples (*p* < 0.05). In each column, different upper-case superscript letters denote significant differences among total SFA, MUFA, and PUFA contents (*p* < 0.05).

**Table 4:** Amino acid profile (in mg/100 g wet weight and mg/g protein) of the muscle proteins of *C. maritae* caught in March and October (average ± standard deviation).

Amino Acids	March		October	
	Amino Acids (mg/100g)	Amino Acids (mg/g Protein)	Amino Acids (mg/100g)	Amino Acids (mg/g Protein)
**Essential**				
Threonine	639 ± 33	36 ± 1	620 ± 118	36 ± 6
Valine	586 ± 41	33 ± 2	597 ± 102	34 ± 6
Methionine	487 ± 34	27 ± 1	453 ± 83	26 ± 4
Isoleucine	579 ±43	32 ± 2	606 ± 112	35 ± 6
Leucine	1167 ± 86	65 ± 4	1152 ± 214	67 ± 12
Phenylalanine	609 ± 40	34 ± 2	611 ± 91	35 ± 5
Histidine	320± 23	18 ± 1	306 ± 51	17 ± 3
Lysine	1096 ± 36	61 ± 2	1069 ± 208	62 ± 12
∑EAA	5486± 339	309 ± 19	5418 ± 982	316 ± 57
**Non-essential**				
Aspartic acid	1706 ± 33	96 ± 1	1627 ± 323	95 ± 19
Serine	664 ± 32	37 ± 1	635 ± 106	37 ± 6
Cysteine	103 ± 13	5 ± 0	87 ± 17	5 ± 0
Glutamic acid	2856 ± 59	161 ± 3	2740 ± 513	160 ± 30
Glycine	1023 ± 95	57 ± 5	979 ± 143	57 ± 8
Alanine	938 ± 36	53 ± 2	887 ± 154	51 ± 9
Tyrosine	601 ± 37	33 ± 2	595 ± 86	34 ± 5
Proline	696 ± 60	39 ± 3	681 ± 112	39 ± 6
Arginine	1377 ± 100	77 ± 5	1309 ± 234	76 ± 13
∑NEAA	9966 ± 470	563 ± 26	9545 ± 1698	558 ± 99
∑TAA	15453 ± 809	873 ± 45	14963 ± 2680	875 ± 156
EAA/NEAA	0.55 ± 0.01	0.55 ± 0.01	0.57 ± 0.02	0.57 ± 0.02

**Table 5:** Amino acid requirements for adults, according to WHO/FAO/UNU [33], and amino acid score (%) of the muscle proteins of *C. maritae* in both seasons (March and October).

Amino Acids	Requirements (mg/g Protein)	Amino Acid Score	
		March	October
Threonine	23	157	157
Valine	39	84	89
Methionine	16	172	165
Isoleucine	30	109	118
Leucine	59	111	114
Phenylalanine + tyrosine	38	179	185
Histidine	15	120	119
Lysine	45	137	138

**Table 6:** Concentration of macro, trace, and toxic elements (mg/100 g wet weight) of the edible tissues of boiled *C. maritae* caught in March and October (average ± standard deviation).

Element		March		October		
		Muscle	Ovaries	Muscle	Ovaries	Hepatopancreas
*Macro*	AI (mg/day)					
Sodium (Na)	1500 ^1,^*	690.63 ± 35.75 ^a^	na	653.24 ± 3.78 ^a^	na	na
Magnesium (Mg)	230–350 ^2^	76.08 ± 5.69 ^a^	na	79.03 ± 1.55 ^a^	na	na
Phosphorous (P)	440–550 ^2^	152.00 ± 5.04 ^a^	na	155.19 ± 1.12 ^a^	na	na
Potassium (K)	1100–3500 ^2^	214.51 ± 16.13 ^a^	na	219.42 ± 1.62 ^a^	na	na
*Trace*						
Iron (Fe)	7–16 ^2^	1.850 ± 0.030 ^a^	na	1.200 ± 0.221 ^a^	na	na
Selenium (Se)	0.020–0.070 ^2^	0.173 ± 0.003 ^a^	na	0.116 ± 0.011 ^b^	na	na
Iodine (I)	0.090–0.150 ^2^	0.147 ± 0.010 ^b^	na	0.187 ± 0.008 ^a^	na	na
Manganese (Mn)	1.0–3.0 ^2^	0.028 ± 0.009 ^c^	0.139 ± 0.001 ^a^	0.024 ± 0.002 ^c^	0.125 ± 0.003 ^b^	0.145 ± 0.002 ^a^
Copper (Cu)	1.0–1.6 ^2^	1.492 ± 0.103 ^c^	2.425 ± 0.224 ^a^	1.506 ± 0.028 ^c^	2.245 ± 0.057 ^b^	2.623 ± 0.062 ^a^
Zinc (Zn)	5.5–16.3 ^2^	4.035 ± 0.002 ^b^	22.048 ± 0.309 ^a^	4.168 ± 0.063 ^b^	19.271 ± 0.549 ^a^	5.484 ± 0.081 ^b^
Nickel (Ni)	NDf ^1^	0.022 ± 0.002 ^c^	0.063 ± 0.011 ^b^	0.019 ± 0.000 ^c^	0.047 ± 0.003 ^b^	0.091 ± 0.001 ^a^
Chromium (Cr)	NDf ^3^	0.006 ± 0.001 ^c^	0.008 ± 0.000 ^c^	0.007 ± 0.000 ^c^	0.014 ± 0.002 ^a^	0.011 ± 0.001 ^b^
Cobalt (Co)	NDf	0.003 ± 0.000 ^d^	0.010 ± 0.001 ^b^	0.002 ± 0.000 ^d^	0.008 ± 0.000 ^c^	0.014 ± 0.000 ^a^
Vanadium (V)	NDf ^1^	0.033 ± 0.001 ^b^	0.044 ± 0.009 ^b^	0.031 ± 0.003 ^b^	0.037 ± 0.000 ^b^	0.070 ± 0.001 ^a^
*Toxic*	MPC (mg/100g)					
Arsenic (As)	NDf	0.109 ± 0.003 ^c^	0.166 ± 0.008 ^b^	0.120 ± 0.005 ^c^	0.162 ± 0.013 ^b^	0.222 ± 0.005 ^a^
Cadmium (Cd)	0.05 ^4^	0.001 ± 0.000 ^a^	<LoD	0.001 ± 0.000 ^a^	<LoD	0.002 ± 0.000 ^a^
Lead (Pb)	0.05 ^4^	0.001 ± 0.000 ^a^	<LoD	0.001 ± 0.000 ^a^	<LoD	0.002 ± 0.000 ^a^
Mercury (Hg)	0.05 ^4^	0.018 ± 0.001 ^a^	0.003 ± 0.000 ^c^	0.018 ± 0.001 ^a^	0.004 ± 0.000 ^c^	0.010 ± 0.001 ^b^

(na) Not analyzed. (LoD) Limit of detection. (MPC) Maximum Permissible Concentration limits. (AI) Adequate Intakes according to EFSA [45]. (NDf) Not defined (*) dietary needs for adults (1) [49], (2) Reference [45], (3) Reference [50], and (4) Reference [51].
